# Risk for sepsis during mechanical circulatory support★

**DOI:** 10.1051/ject/2025041

**Published:** 2025-12-17

**Authors:** Kelsey Gore, Dean Linder, Juan José Martinez Duque, Junxi Wang, Joshua Baguley, Cortland Kolesnikowicz, Shaun Yockelson, Manila Singh, Adrian Alexis Ruiz, Bobby D. Nossaman

**Affiliations:** 1 Department of Cardiovascular Perfusion and Extracorporeal Technology, Ochsner Health 1514 Jefferson Highway New Orleans Louisiana 70121 USA; 2 CES University Cl 10A #22-04 El Poblado, Medellin Antioquia Colombia; 3 The University of Queensland Medical School 288 Herston Road Herston QLD 4006 Australia; 4 Department of Critical Care Section, Anesthesiology & Perioperative Medicine, Ochsner Health 1514 Jefferson Highway New Orleans Louisiana 70121 USA

**Keywords:** Sepsis, Mechanical circulatory support (MCS), Congestive heart failure (CHF), Cardiac surgery, Cardiovascular disease, Inflammation

## Abstract

*Introduction*: Patients receiving mechanical circulatory support (MCS) risk the development of sepsis. Examining risk factors for the development of sepsis and their relationships to MCS may allow for an improved understanding of these complications. *Methods*: Following IRB approval, patient characteristics, previously reported comorbidities, and the incidence of sepsis were studied in 199 patients who received 244 MCS therapies from January 2017 to October 2023. The clinical variables underwent ensemble machine learning modeling. Significant comorbidities predicting sepsis from the ensemble machine modeling underwent decision-tree analysis. *Results*: In this study, the incidence of sepsis was 20% (95% CI: 16–26%). Following machine learning modeling, patients with a history of congestive heart failure or a history of previous cardiac surgery were associated with an increased risk for developing sepsis. The c-index statistic for this model was 0.76, with a misclassification rate of 19%. Decision-tree analysis observed that patients without chronic cardiovascular disease but with a history of prior cardiac surgery have a 60.3% (95% CI: 60.1–65.2%) incidence of sepsis during MCS therapy. Patients with a history of chronic cardiovascular disease and with a history of congestive heart failure have an 18.1% (95% CI: 17.2–18.7%) incidence of developing sepsis. *Conclusion*: The incidence of sepsis is high in this patient population. The novel associations of patients who have histories of congestive heart failure or previous cardiac surgery requiring MCS suggest an increased systemic inflammatory state exists that escalates the risk for developing sepsis. Further investigation into these background inflammatory conditions in patients requiring MCS is warranted.

## Introduction

Mechanical circulatory support (MCS) devices are advanced treatment options for patients with refractory cardiopulmonary failure [1–6]. These devices can be categorized into two classes: acute and durable [7]. Acute MCS devices are temporary therapies for refractory cardiopulmonary failure. Acute MCS devices include intra-aortic balloon pumps (IABPs), Impella devices, extracorporeal membrane oxygenation (ECMO), extracorporeal ventricular assist devices (VADs) such as Centrimags, and TandemHeart devices (Appendix). MCS devices serve as treatment for transplantation, or if transplantation is not an option, as a destination therapy. The durable and/or destination devices implanted were Heartmate VADs. Whether acute or durable, patients requiring MCS therapy are subject to many complications, such as hemorrhagic, thrombotic, and infections [1, 3].

Patients who receive MCS therapy are critically ill and at a heightened risk of developing sepsis. The invasive nature of the MCS devices, surgical implantation, foreign objects from the extracorporeal circuits, and longer hospital duration are a few factors that increase the risk [1, 7, 8]. An exacerbated inflammatory response from the administration of MCS devices creates a complex pathophysiology [8]. Sepsis is a potentially life-threatening condition brought on by a dysregulated physiological response to an infection [9]. What is known is that patients who require MCS are critically ill and susceptible to many complications, whether from their disease, invasive procedures, or the devices themselves [1]. Infection is a known and previously documented complication for MCS patients [1, 3, 4]. However, the cause of sepsis in patients undergoing MCS requires further investigation into the specific devices, duration of devices, and which specific preprocedural comorbidities exacerbate their risk. In our study, we aim to gain a better understanding of preprocedural comorbidities in patients undergoing MCS and developing sepsis. With this, clinical decision-making could be improved for these critically ill patients.

## Materials and methods

Following IRB approval, a retrospective analysis of preprocedural patient comorbidities (Table 1) and the incidence of sepsis was recorded from January 2017 to October 2023 [2, 4, 10]. The study included 199 patients who received 244 (Appendix) MCS therapies at Ochsner Health-Jefferson Highway Campus in New Orleans, Louisiana. In terms of durable VADs, the data collected was focused on the post-operative acute phase (post-operative day (POD) 0 to day 14 following implantation) to support better the data collected from the short-term MCS devices. Patients aged 18 years or older who received MCS were included in this study. There were no patient exclusion criteria.

**Table 1:** Contingency table of the association of sepsis to congestive heart failure in 199 Patients receiving 244 mechanical circulatory support devices.

		Sepsis		
	Counts (%)	Yes	No	Totals
Congestive heart failure	Yes	32 (23)	109 (77)	141
	No	17 (17)	86 (84)	103
	Totals	49	195	244

To identify risk factors, previously reported comorbidities and patient characteristics, age, sex, body mass index (BMI), the comorbidities of insulin-dependent diabetes, chronic renal failure, chronic cardiovascular disease, immunomodulation, structural lung disease, ICD/Pacemaker, atrial fibrillation, endocarditis, previous cardiac surgery, congestive heart failure, and peripheral vascular disease were placed in predictor models. In our cohort of patients, the diagnostic workup for sepsis included clinical assessments for fever, tachycardia, or tachypnea, including signs of organ dysfunction (hypotension, altered mental status, temperature). Laboratory tests such as complete blood count (CBC), lactate levels, kidney and liver function, arterial blood gases (ABGs), and blood and urine cultures.

### Statistics

**Figure 1:**
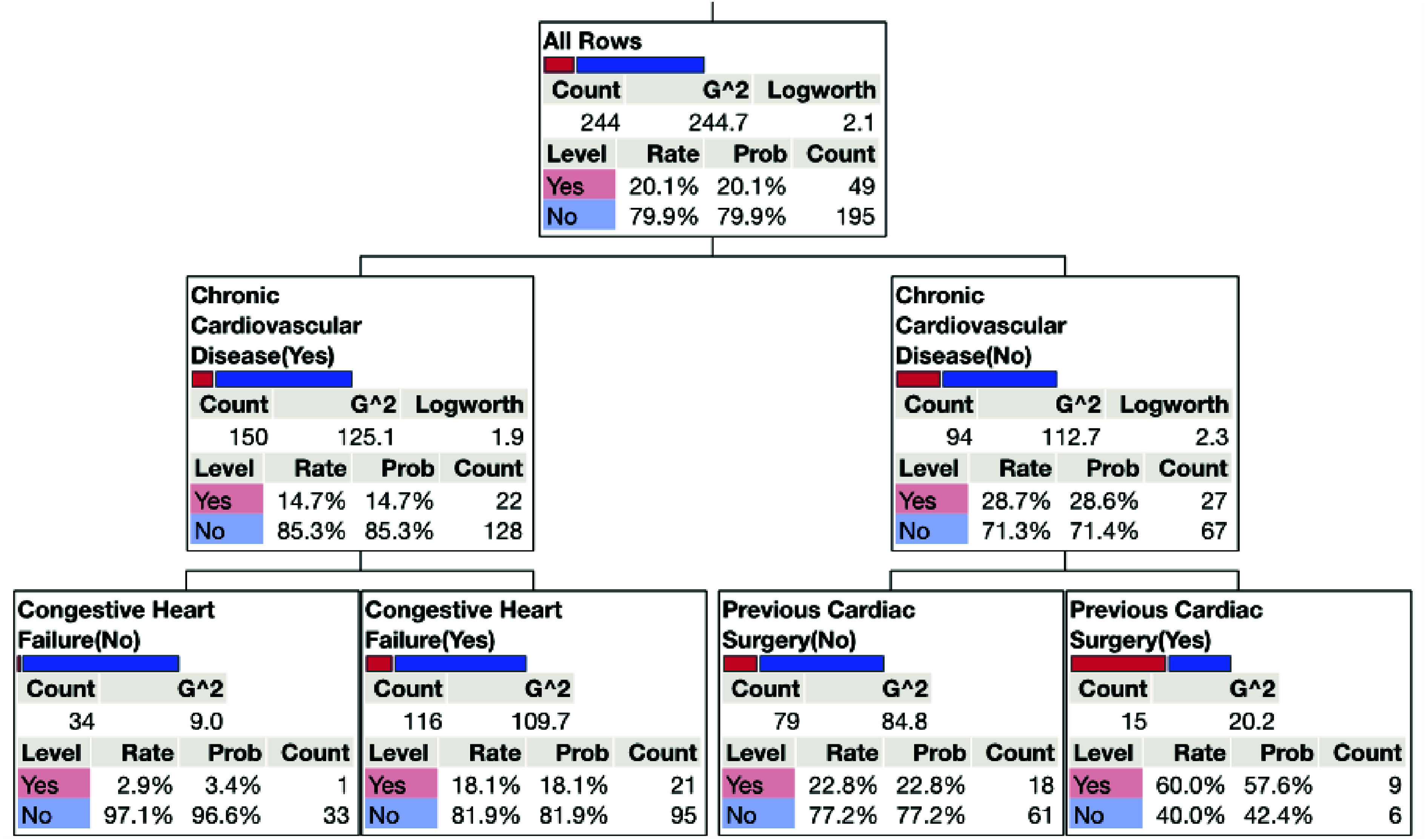
Decision tree analysis of significant comorbidities and sepsis incidence.

Our study used ensemble machine learning analysis to identify those comorbidities (Tables 1 and 2) that may predict the development of sepsis [11]. Furthermore, decision tree analysis (Figure 1) was performed to clarify the relationships between these factors and the incidence of sepsis [12]. Identification of preprocedural patient comorbidities and the incidence of sepsis was performed using JMP Pro 18.2 (SAS, Inc., Cary, NC).

**Table 2:** Contingency table of the association of sepsis to previous cardiac surgery in 199 patients receiving 244 mechanical circulatory support devices.

		Sepsis		
	Counts (%)	Yes	No	Totals
Previous cardiac surgery	Yes	21 (26)	60 (74)	81
	No	28 (17)	135 (83)	163
	Totals	49	195	244

## Results

In this study, the incidence of sepsis was 20% CI (16–26%). Following ensemble machine learning analysis of the patient characteristics and the 14 comorbidities, patients with a history of congestive heart failure (CHF) or a history of previous cardiac surgery were associated with an increased risk for developing sepsis (Tables 1 and 2). The c-index statistic for this model was 0.76 with a misclassification rate of 19%.

We further explored these two significant preprocedural comorbidities, congestive heart failure and previous cardiac surgery. This analysis observed that patients without a history of cardiovascular disease (CAD) but with a history of prior cardiac surgery have a 60.3% CI (60.1–65.2%) incidence of sepsis during MCS therapy (Figure 1). However, patients with a history of CAD and CHF have an 18.1% CI (17.2–18.7%) incidence of developing sepsis during MCS therapy (Figure 1).

## Discussion

This study observed that patients with a previous history of CHF or previous open-heart surgery had an increased risk for developing sepsis. Congestive heart failure contributes to increased hospital admissions due to worsening outcomes. Sepsis is associated with increased mortality in CHF patients [13]. This comorbidity of patients requiring MCS who develop sepsis has a complex interplay. Congestive heart failure patients, specifically with lower ejection fractions, live in a proinflammatory state that is like that of cardiogenic shock [13]. Sepsis in the failing heart increases nitric oxidase synthase (NOS), leading to reduced systolic calcium levels, in turn reducing cellular contraction. Treating sepsis in patients requiring MCS with CHF becomes a balancing act to not overload the cardiovascular system [13, 14]. This complex pathophysiology warrants further investigation into CHF MCS patients’ risk factors and further understanding of sepsis.

Patients requiring MCS with a pre-existing history of open-heart surgery were found to have an increased risk of sepsis within this patient cohort. Whether this is a recent or past surgery, scar tissue can become a nidus for infection, particularly in poorly perfused scar tissue. It should be noted that our 20% incidence of sepsis is comparable to previous studies, which reported an 18% incidence of sepsis during MCS [4]. This study finds that the high incidence of sepsis highlights the need for improved risk identification and continued research into the complex relationship of sepsis with MCS.

## Limitations

Limitations within this study include data collection completeness due to the retrospective nature of the study and that it is a single-center study. However, the development of electronic medical records has strengthened the data collection process. To further enhance the study from these limitations was the statistical method applied to all confounders with the use of machine model learning algorithms to assess risks, as they reveal information on the associations of clinical significance. Machine learning ensemble is a powerful tool used to express information and outperforms traditional statistical analyses when analyzing multifaceted datasets.

## Conclusions

The incidence of developing sepsis is high in this patient population. The novel associations of patients who have previous comorbidities of CHF or previous cardiac surgery requiring MCS therapy suggest an increased systemic inflammatory state. Further investigation into this proposed mechanism of sepsis and background inflammatory conditions in patients requiring MCS therapy is warranted.

## Data Availability

All available data are incorporated into the article.

## Appendix

**Table A1:** Distribution of acute and durable mechanical circulatory devices.

Acute MCS devices	Count
IABP*	63
Impella	14
TandemHeart	42
ECMO*	100
BiVAD*	3
RVAD*	4

Durable MCS device	Count

LVAD*	18
Total	244

*Intra-aortic balloon pump; Extracorporeal Membrane Oxygenation; Biventricular assist device; Right ventricular device; Left ventricular device, respectively.
